# Functional Outcomes and Quality of Life Following Supraclavicular Flap Reconstruction in Oral Cancer Surgery: A Retrospective Analysis

**DOI:** 10.7759/cureus.83445

**Published:** 2025-05-04

**Authors:** Rachana J, S.M. Azeem Mohiyuddin, Sagayaraj A, Kouser Mohammadi, Ujval Gowda

**Affiliations:** 1 Otolaryngology-Head and Neck Surgery, Sri Devaraj Urs Academy of Higher Education and Research, Kolar, IND; 2 Otolaryngology-Head and Neck Surgery, Sri Devaraj Urs Medical College, Kolar, IND

**Keywords:** functional outcomes, head and neck reconstruction, oral cavity cancer, quality of life, squamous cell carcinoma, supraclavicular flap, surgical flaps

## Abstract

Background and objective

Oral cavity malignancies pose considerable challenges in surgical management, making optimal functional and aesthetic reconstruction crucial after tumor ablation. The supraclavicular flap (SCF) has gained popularity in head and neck reconstruction due to its reliable vascularity, adequate surface area, pliability, and low donor site morbidity. While evidence supporting its use in oral reconstruction is growing, systematic evaluation of functional outcomes and quality of life remains limited. This study aims to assess functional outcomes and quality of life following SCF reconstruction in patients with T2-T3 oral cavity malignancies over a 10-year period.

Methodology

This retrospective study was conducted at a rural tertiary care center, analyzing data from October 2014 to November 2024. A total of 40 patients with biopsy-confirmed squamous cell carcinoma involving the buccal complex and floor of the mouth who underwent SCF reconstruction were included. Data collected included demographics, tumor characteristics, surgical details, postoperative complications, and functional outcomes. Functional assessments focused on speech intelligibility, swallowing capacity, oral competence, and shoulder mobility at three, six, and 12 months postoperatively. Quality of life was evaluated using the World Health Organization Quality of Life-BREF questionnaire at six and 12 months. Statistical analysis was performed using paired t-tests for continuous variables and Cochran’s Q test for categorical data, with significance set at p < 0.05.

Results

The study included 40 patients with a mean age of 54.7 years; the majority were female (67.5%). The buccal mucosa was the most common tumor site (80%), and 57.5% of patients presented with T2 lesions. The average operative time was 95 minutes, and the mean hospital stay was 8.4 days. Flap-related complications occurred in 35% of patients, with partial flap necrosis being the most common (17.5%). Speech intelligibility improved significantly from 64.5% at three months to 86.7% at 12 months (t = 7.92, p < 0.001). Swallowing function improved from a mean Functional Oral Intake Scale score of 4.1 to 6.1 over the same period (t = 9.45, p < 0.001). By 12 months, 82.5% of patients achieved full oral intake without restrictions. Shoulder abduction improved from 121.7° to 157.9° (t = 10.32, p < 0.001). Quality-of-life scores significantly improved between six and 12 months in physical health (59.6 to 68.5, t = 5.78, p < 0.001), psychological well-being (61.3 to 67.9, t = 2.46, p = 0.018), and overall quality of life (61.8 to 68.3, t = 2.89, p = 0.006). At 12 months post-surgery, 62.5% of patients demonstrated excellent functional outcomes, and 15% had good outcomes.

Conclusions

The SCF is an axial fasciocutaneous flap that offers reliable reach to oral cavity defects with minimal donor site morbidity. It is a practical and effective reconstructive option for T2-T3 oral cancers, offering comparable quality-of-life outcomes to microvascular free flaps. The SCF is easy to harvest, pliable, and has dependable vascularity. It also reduces operative time and financial burden, making it particularly beneficial for patients at risk due to long anesthesia duration, peripheral vascular disease, or major comorbidities.

## Introduction

Oral cavity malignancies present significant challenges in surgical management, with the primary objectives being complete oncological resection with adequate margins and optimal functional and aesthetic reconstruction [1]. Given the complex anatomy of the oral cavity and its essential roles in speech, mastication, and swallowing, meticulous reconstructive strategies are crucial following tumor ablation [2]. Various reconstructive techniques have been employed, including local flaps, axial flaps - most commonly the pectoralis major myocutaneous flap - and microvascular free tissue transfer [3].

The supraclavicular flap (SCF) is an axial fasciocutaneous flap based on the supraclavicular branch of the transverse cervical artery. It has become increasingly utilized in the reconstruction of oral cavity cancers, particularly for T2 and T3 defects involving the buccal mucosa, floor of mouth, lower alveolus, and smaller pharyngeal defects. The SCF offers several advantages: reliable vascularity, a generous surface area, pliability, ease and speed of harvest, and minimal donor site morbidity. Additionally, it significantly reduces operative time compared to microvascular free tissue reconstruction [4].

In reconstructing T2-T3 oral cancer defects, the SCF serves as a viable alternative to traditional free flaps such as the radial forearm free flap (RFFF), especially in specific clinical settings [5]. The constraints imposed on healthcare systems during the COVID-19 pandemic further underscored the value of the SCF. As noted by Thompson et al. (2020), its use helped reduce operative time and decreased the need for intensive care resources during the pandemic era [6].

The versatility of the SCF extends to several anatomical subsites within the oral cavity, including the buccal mucosa, floor of mouth, and tongue [7,8]. Multi-institutional studies have shown favorable outcomes in terms of flap survival, wound healing, and donor site recovery [9-11]. Technical considerations, such as careful preservation of the vascular pedicle and appropriate tunneling to the recipient site, have been well described in the literature [12].

Despite the growing body of evidence supporting SCF use in oral reconstruction, systematic evaluations of functional outcomes and quality of life remain limited. While studies like that of Spiegel et al. (2019) have compared quality of life between SCF and RFFF, comprehensive assessments using standardized instruments such as the World Health Organization Quality of Life-BREF (WHOQOL-BREF) remain underreported in the context of SCF reconstruction for oral cancer defects [5].

The functional outcomes of SCF reconstruction, particularly related to speech and swallowing, require thorough investigation, given their significant impact on patient satisfaction and overall post-treatment quality of life [13]. Moreover, potential donor site issues such as shoulder mobility limitations, paresthesia, and cosmetic concerns must be carefully considered to guide surgical decision-making [14].

This study aims to evaluate the functional outcomes and quality of life following SCF reconstruction in patients with T2-T3 oral cavity malignancies. By analyzing a 10-year cohort, it seeks to provide meaningful evidence on the effectiveness of the SCF in preserving essential oral functions while ensuring satisfactory oncological control and overall quality of life.

## Materials and methods

Study design and study setting

This retrospective study was conducted at a tertiary care center in a rural region, equipped with a dedicated head and neck oncology unit. A retrospective design was chosen to enable a comprehensive evaluation of functional outcomes and quality of life in patients who underwent SCF reconstruction following oral cancer surgery.

Study period

The study covered a 10-year period, from October 2014 to November 2024. This extended timeframe was selected to ensure an adequate sample size and to facilitate the assessment of long-term functional outcomes.

Ethics committee approval

The study protocol received approval from the Institutional Ethics Committee (approval number SDUAHER/KLR/R&D/CEC/S/PG/89/2024-25) prior to data collection. All procedures adhered to the ethical standards of the institutional research committee and the principles outlined in the 1964 Helsinki Declaration and its later amendments. Due to the retrospective nature of the study, a waiver of informed consent was granted. However, all patient data were anonymized and coded to ensure confidentiality.

Inclusion criteria

Patients were included if they were between 30 and 70 years of age, had biopsy-proven squamous cell carcinoma involving the buccal complex and/or floor of the mouth, and underwent composite resection with SCF reconstruction at the institution between October 2014 and November 2024.

Exclusion criteria

Exclusion criteria included a history of prior neck or supraclavicular surgery that compromised the vascular supply to the SCF, severe shoulder dysfunction or comorbidities likely to affect flap harvesting or functional evaluation, previous radiation to the neck region or preexisting neck contractures, and documented peripheral vascular disease.

Sample size estimation

The sample size was calculated using Cochran’s formula:

\[ n = \frac{Z^2 \cdot p \cdot (1 - p)}{d^2}, \]

where Z is the standard normal variate (1.96 at a 5% level of significance), p is the expected proportion, and d is the absolute precision.

Based on prior studies, Sandu et al. (2012) reported that 18% of patients experienced significant functional limitations after SCF reconstruction in a cohort of 50 patients [9], while D’Aco et al. (2023) reported a functional impairment rate of 22% in a group of 37 patients [11]. Using the higher prevalence value of 22%, an absolute precision of 15%, and a 95% confidence level, the minimum required sample size was estimated at 36 patients. To accommodate potential data loss or incompleteness, the final target sample size was set at 40 patients.

Sampling method

A consecutive sampling method was used, wherein all eligible patients who underwent SCF reconstruction for oral cancer during the study period were included until the target sample size was reached.

Data collection procedure

**Figure 1:**
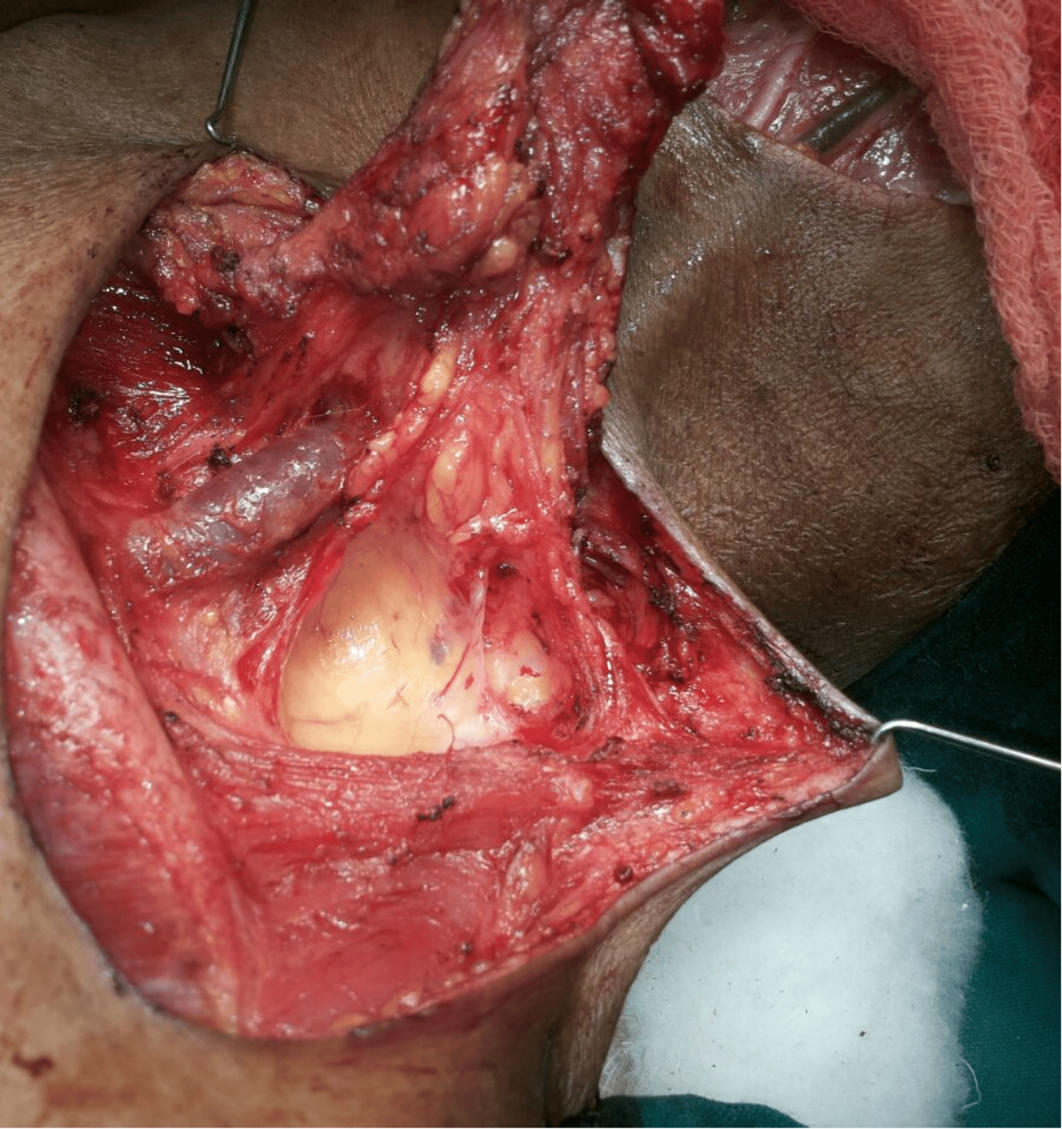
Intraoperative raising of the SCF This image demonstrates the elevation of the supraclavicular island flap based on the transverse cervical artery branch. SCF, supraclavicular flap

Data collection was performed through a thorough review of patient medical records, operative notes, and follow-up documentation. A standardized data extraction form was employed to ensure consistency and accuracy. Preoperative variables included patient demographics, comorbidities, and tumor characteristics such as site, size, and TNM staging. Intraoperative data included the surgical approach, flap dimensions, and details of the recipient site. Postoperative variables recorded were duration of hospital stay, complications such as partial or total flap necrosis, wound infection, or chyle leak, as well as the timeline of functional recovery. Figure 1 shows the raising of the SCF.

Functional outcomes were assessed using a structured questionnaire designed to evaluate speech intelligibility, swallowing capacity, oral competence, and shoulder mobility. This questionnaire was adapted from validated instruments previously used by Patro et al. (2019) and Modi (2022) [2,15]. Its content validity was established through peer review by a panel of five experienced head and neck surgeons prior to its implementation. A pilot study with 10 patients was conducted to assess the feasibility and reliability of the questionnaire, yielding a Cronbach’s alpha of 0.87, which indicates good internal consistency.

Composite functional outcome assessment framework

**Figure 2:**
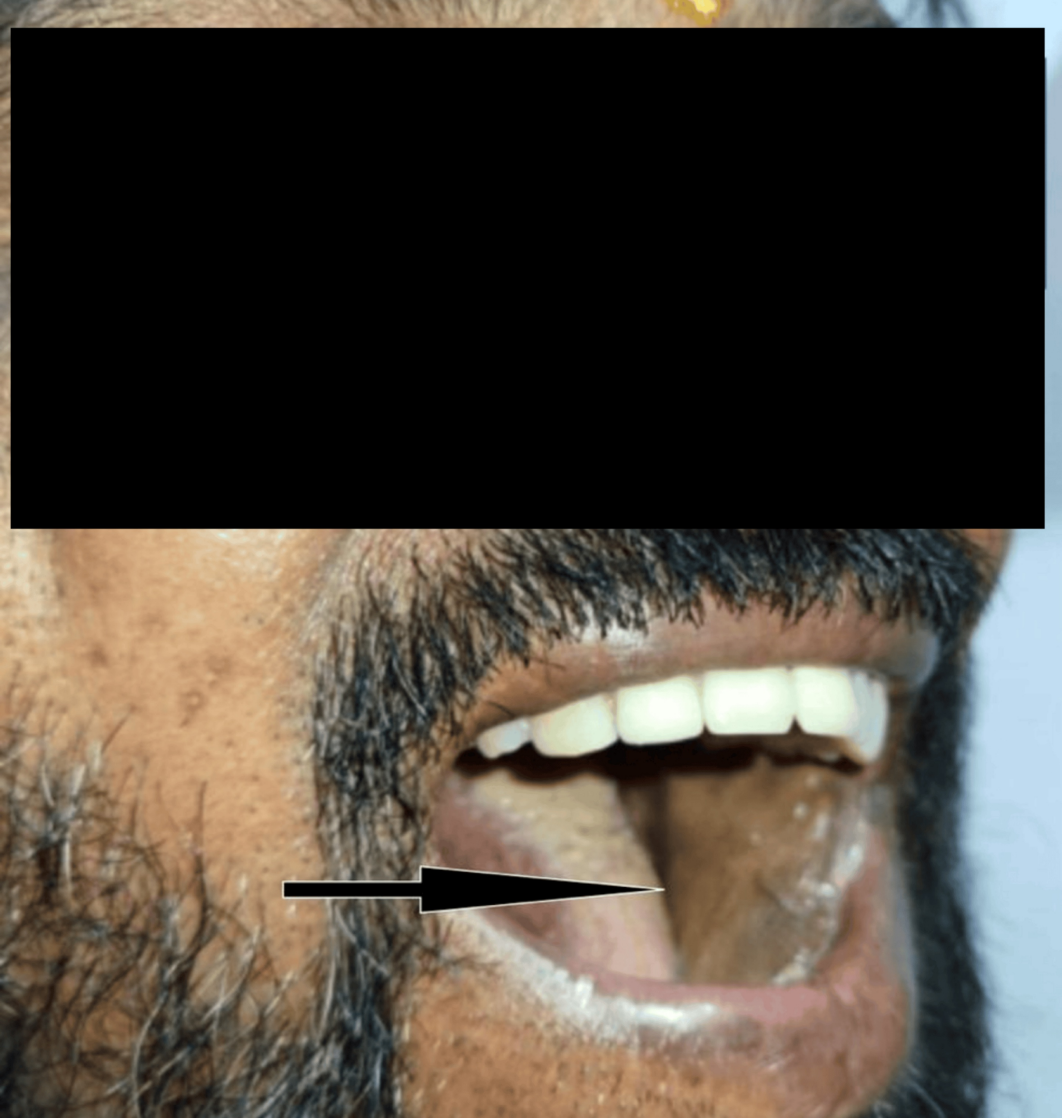
Postoperative intraoral flap uptake This image illustrates the successful integration of the SCF into the buccal mucosa defect site, with good mucosal uptake observed. SCF, supraclavicular flap

**Figure 3:**
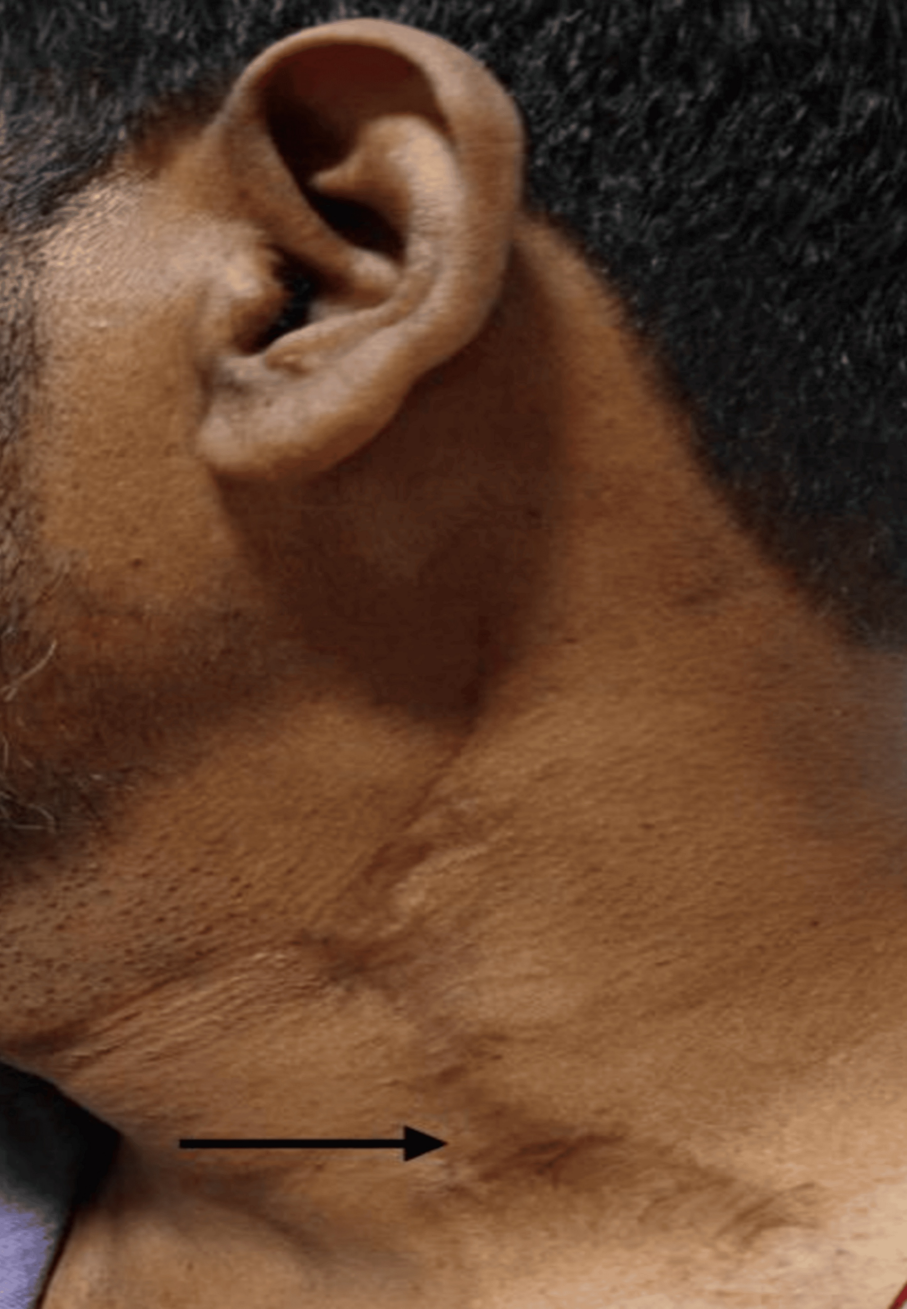
Healed donor site scar over the supraclavicular region A 12-week postoperative view showing complete healing of the donor site, with a cosmetically acceptable linear scar in the supraclavicular area.

A composite functional outcome score was calculated based on weighted values assigned to key functional domains: speech intelligibility (30%), swallowing ability (30%), mouth opening (20%), and oral intake status (20%). These weightings reflect the relative clinical importance of each parameter in evaluating overall functional recovery following oral reconstruction. Speech and swallowing were given greater proportional significance due to their essential role in maintaining oral function. Postoperative integration of the SCF into the intraoral defect is illustrated in Figure 2, while the healed donor site scar is shown in Figure 3.

Outcome classification system

Based on the composite functional outcome score, patients were categorized into three distinct groups at 12 months post-surgery: excellent outcome (composite score ≥80%), good outcome (60-79%), and poor outcome (<60%). This classification allowed for a standardized assessment of recovery following SCF reconstruction.

Quality of life was assessed using the WHOQOL-BREF questionnaire, a validated tool for head and neck cancer patients, including in studies such as Bakshi et al. (2022) [1]. The WHOQOL-BREF evaluates four domains: physical health, psychological well-being, social relationships, and environmental support. Patients completed the questionnaire during follow-up visits, with a minimum follow-up duration of six months to ensure adequate postoperative recovery and adjustment.

Data analysis

Statistical analysis was conducted using IBM SPSS Statistics for Windows, Version 26.0 (Released 2019; IBM Corp., Armonk, NY, USA). Descriptive statistics were used to summarize demographic and clinical variables, with categorical data presented as frequencies and percentages, and continuous data as means with SDs. Functional outcome scores at three, six, and 12 months post-surgery were compared using repeated-measures ANOVA, followed by Bonferroni-adjusted post hoc tests. T-values were calculated to compare outcomes between three and 12 months. Categorical variables, such as oral intake status, were analyzed using Cochran’s Q test. Quality-of-life scores between six and 12 months were compared using paired t-tests. Statistical significance was set at p < 0.05, with CIs calculated at the 95% level.

## Results

The study included 40 patients, with a mean age of 54.7 years (SD = 9.3). Of these, 27 (67.5%) were female, and 13 (32.5%) were male. Comorbidities were present in 30 patients (75%), with hypertension being the most common, reported in 15 individuals (37.5%). A significant proportion of patients (82.5%) reported addiction to chewable tobacco and betel nut quid. Based on the American Society of Anesthesiologists (ASA) physical status classification, eight patients (20.0%) were categorized as ASA I.

The buccal mucosa was the most common tumor site, observed in 32 patients (80%), followed by the floor of the mouth in eight patients (20%). With respect to tumor staging, 23 patients (57.5%) presented with T2 lesions. Clinically, the majority - 27 patients (67.5%) - had no nodal involvement (N0), while three patients (7.5%) were staged as N1 and 10 (25%) as N2. The mean flap dimension used for reconstruction was 6.7 cm² (SD = 1.5), and the average flap thickness was 7.2 mm (SD = 1.6). The mean operative time was 95 minutes (SD = 12.5), and the average postoperative hospital stay was 8.4 days (SD = 3.2).

Table 1 summarizes the postoperative complications and clinical outcomes following SCF reconstruction. No flap-related complications were observed in 26 patients (65%). Among those who did experience complications, partial flap necrosis was the most common, occurring in seven patients (17.5%). Donor site morbidity was absent in 24 patients (60%), while paresthesia was the most frequently reported complication at the donor site, affecting 11 patients (27.5%). Systemic complications were minimal, with pneumonia reported in only two patients (5.0%). All patients received adjuvant therapy: 27 (67.5%) underwent radiotherapy alone, while 13 (32.5%) received chemoradiotherapy. Phantom pain was reported by 10 patients (25%).

**Table 1:** Postoperative complications and outcomes

Complication/outcome	N (%)
Flap-related complications	
No complications	26 (65%)
Complete flap necrosis	2 (5.0%)
Partial flap necrosis	7 (17.5%)
Wound infection	5 (12.5%)
Wound dehiscence	4 (10.0%)
Oro-cutaneous fistula formation	2 (5.0%)
Donor site complications	
No complications	24 (60%)
Wound infection	2 (5.0%)
Wound dehiscence	3 (7.5%)
Hypertrophic scarring	6 (15.0%)
Paresthesia	11 (27.5%)
Phantom pain	10 (25.0%)
Systemic complications	
Pneumonia	2 (5.0%)
Mortality within 30 days	0 (0.0%)

Table 2 presents the functional outcomes assessed at sequential time points following SCF reconstruction in patients with oral cancer. A consistent and significant improvement was observed across all functional domains over the 12-month postoperative period. Speech intelligibility improved markedly from 64.5% at baseline to 86.7% at 12 months. Swallowing ability, measured by the Functional Oral Intake Scale, showed an increase from a mean score of 4.1 to 6.1. Mean mouth opening also expanded significantly, from 22.6 mm to 34.2 mm. Additionally, neck rotation improved from 57.3° to 75.1°, while shoulder abduction increased from 121.7° to 157.9°. Oral intake capacity demonstrated substantial gains, with the proportion of patients able to resume full oral intake without dietary restrictions rising from 32.5% to 82.5%. Notably, by six months post-surgery, all patients who had previously required partial tube feeding had successfully transitioned to complete oral intake.

**Table 2:** Functional outcomes at different time points post-surgery * p-value refers to comparisons across all time points: repeated-measures ANOVA with Bonferroni-adjusted post-hoc t-tests was used for continuous variables (values reported for three-month vs. 12-month comparisons), while Cochran’s Q test (χ² values) was used for categorical variables related to oral intake status. FOIS = Functional Oral Intake Scale (1-7, higher score indicates better function)

Function	Three months (mean ± SD)	Six months (mean ± SD)	12 months (mean ± SD)	Test statistic	p-value*
Speech intelligibility (%)	64.5 ± 18.7	78.3 ± 15.2	86.7 ± 10.4	t = 7.92	<0.001
Swallowing ability (FOIS score)	4.1 ± 1.2	5.3 ± 1.0	6.1 ± 0.8	t = 9.45	<0.001
Mouth opening (mm)	22.6 ± 8.4	29.4 ± 7.2	34.2 ± 6.1	t = 8.13	<0.001
Neck rotation (degrees)	57.3 ± 14.5	67.8 ± 11.3	75.1 ± 9.5	t = 7.26	<0.001
Shoulder abduction (degrees)	121.7 ± 22.6	142.5 ± 18.4	157.9 ± 13.7	t = 10.32	<0.001
Oral intake status					
Full oral intake without restrictions, n (%)	13 (32.5)	27 (67.5)	33 (82.5)	χ² = 26.37	<0.001
Soft diet, n (%)	19 (47.5)	10 (25.0)	5 (12.5)	χ² = 18.82	<0.001
Liquid diet, n (%)	6 (15.0)	3 (7.5)	2 (5.0)	χ² = 12.44	<0.001
Partial dependency on tube feeding, n (%)	2 (5.0)	0 (0.0)	0 (0.0)	χ² = 9.86	0.002

Table 3 presents the quality-of-life outcomes assessed using the WHOQOL-BREF at six and 12 months following SCF reconstruction. Significant improvements were observed across multiple domains over the follow-up period. Physical health scores increased from 59.6 to 68.5, psychological well-being improved from 61.3 to 67.9, and social relationship scores rose from 66.5 to 70.7. Although the environmental domain showed a modest increase from 64.3 to 66.9, this change was not statistically significant. Overall quality of life and general health perception improved significantly, from 61.8 at six months to 68.3 at 12 months, indicating substantial recovery and enhanced well-being in most domains by the end of the study period.

**Table 3:** Quality-of-life outcomes assessed by WHOQOL-BREF at six and 12 months following SCF reconstruction (n = 40) * p-value refers to the comparison between six-month and 12-month scores using a paired t-test. WHOQOL-BREF scores range from 0 to 100, with higher scores indicating better quality of life. SCF, supraclavicular flap; WHOQOL-BREF, World Health Organization Quality of Life-BREF

Domain	Six months (mean ± SD)	12 months (mean ± SD)	t-value	p-value*
Physical health	59.6 ± 16.8	68.5 ± 14.2	5.78	<0.001
Psychological well-being	61.3 ± 17.5	67.9 ± 15.3	2.46	0.018
Social relationships	66.5 ± 15.4	70.7 ± 14.1	2.1	0.042
Environment	64.3 ± 16.1	66.9 ± 15.3	0.93	0.357
Overall quality of life and general health	61.8 ± 17.2	68.3 ± 15.5	2.89	0.006

Table 4 presents the composite functional outcome assessment at 12 months following SCF reconstruction in patients with oral cancer. At the 12-month mark, 25 patients (62.5%) achieved excellent outcomes, six patients (15.0%) had good outcomes, and nine patients (22.5%) were classified as having poor outcomes based on the composite score.

**Table 4:** Overall functional outcome score at 12 months post-surgery

Functional outcome category	N (%)
Excellent outcome	25 (62.5%)
Good outcome	6 (15.0%)
Poor outcome	9 (22.5%)
Total	40 (100.0%)

Functional outcome categories were derived from a composite assessment of speech intelligibility, swallowing ability, mouth opening, and oral intake status, following the scoring system described in the methodology.

## Discussion

In our study, the buccal mucosa was the most common subsite involved (80%), consistent with findings from other studies in South Asia. Ranjan et al. (2024) similarly reported that buccal mucosa was the predominant site in 72% of their 78 patients in India [7]. Our cohort excluded T4 tumors involving skin, focusing instead on T2 (57.5%) and T3 (42.5%) lesions - an appropriate selection for SCF reconstruction, as supported by literature [5,16].

SCF-related complications occurred in 35% of cases, although complete flap necrosis was limited to just 5%, mainly in the early part of the study when the external jugular vein was routinely sacrificed. Preservation of venous drainage into the external jugular vein significantly reduced flap failure rates. Sandu et al. (2012) reported a similar complete flap necrosis rate of 4% in their series of 50 patients [9]. Likewise, D’Aco et al. (2023) documented a 32% complication rate in their study of 37 Italian patients, with partial necrosis being the most common complication [11]. These failures can stem from technical issues, poor patient selection, nutritional status, infections, or postoperative fistula formation.

A notable strength of this study is the longitudinal evaluation of functional outcomes at three, six, and 12 months. Speech intelligibility improved markedly from 64.5% at three months to 86.7% at 12 months, reflecting significant functional recovery. Swallowing function also improved substantially, with 82.5% of patients achieving full oral intake without restrictions by 12 months. These results align with those reported by Spiegel et al. (2019), who found comparable outcomes at 12 months between SCF (n = 21) and RFFF (n = 22) groups, suggesting that SCF can provide similar functional results to RFFF in well-selected cases [5].

The increase in mouth opening from 22.6 mm at three months to 34.2 mm at 12 months is clinically significant, as it impacts oral intake, dental hygiene, and overall quality of life. Our findings are consistent with those of Klibngern et al. (2024), who reported a mean mouth opening of 32.8 mm at 12 months in a cohort of 28 patients undergoing SCF reconstruction for oral tongue cancer in Thailand [8]. Shoulder function also improved significantly, with shoulder abduction increasing from 121.7° to 157.9°, particularly after targeted physiotherapy. However, this contrasts with Zaid et al. (2022), who found persistent shoulder dysfunction in 22% of 32 patients in Egypt at 12 months [14], possibly due to variations in surgical technique or flap size.

Quality-of-life outcomes, assessed using the WHOQOL-BREF, showed significant improvement across multiple domains between six and 12 months. The physical health domain exhibited the most notable gain (from 59.6 to 68.5, p < 0.001), whereas the environmental domain showed only minimal change (64.3 to 66.9, p = 0.357). These results echo those of Spiegel et al. (2019), who used the University of Washington Quality of Life questionnaire and reported similar outcomes between SCF and RFFF [5]. Our findings are also in line with Molnár et al. (2022), who observed similar trajectories in physical health improvement in their series of 107 head and neck cancer patients in Finland [13].

The SCF offers additional advantages, such as shorter operative time and the feasibility of a single-team approach, which can help reduce financial burden, especially important for patients with significant comorbidities or anesthesia-related risks. The significant increase in overall quality of life and general health perception (from 61.8 to 68.3, p = 0.006) reinforces the utility of SCF in selected patients.

At 12 months, 62.5% of our patients achieved excellent functional outcomes, and 15.0% achieved good outcomes, underscoring the efficacy of SCF in oral cavity reconstruction. The 22.5% with poor outcomes likely reflect more complex defects, advanced disease stages, or complications that hindered recovery. This distribution closely mirrors the 24% suboptimal outcome rate reported by Thaduri et al. (2023) in their SCF cohort of 46 patients in India [10].

The comparable quality-of-life outcomes between SCF and free flap reconstructions suggest that free flaps may not always confer superior patient-reported benefits. SCF is particularly beneficial for patients with high anesthetic risk, peripheral vascular disease, or other major comorbidities.

Limitations

This study has several limitations that should be considered when interpreting its findings. Its retrospective design inherently introduces the potential for selection and recall bias, particularly in the assessment of subjective parameters. As a single-institution study with a limited sample size, the analysis lacks comparison with free flap reconstruction. Additionally, the evaluation of functional outcomes at fixed intervals (three, six, and 12 months) may not fully capture the variability in individual recovery patterns or long-term functional changes beyond the 12-month period. The potential impact of adjuvant therapy on functional outcomes and quality of life was not controlled for, although all patients received either radiotherapy (67.5%) or chemoradiotherapy (32.5%). These treatments are known to affect tissue healing, fibrosis, and functional recovery and may therefore confound the assessment of outcomes specific to the reconstruction method.

## Conclusions

The SCF is an axial fasciocutaneous flap with sufficient reach for oral cavity defects, resulting in minimal donor site morbidity. It serves as an effective reconstructive option following resection of T2 and T3 oral cancers and offers quality-of-life outcomes comparable to those achieved with microvascular free tissue transfer. The flap is easy to harvest, pliable, and reliably vascularized. It also reduces operative time and financial burden, making it particularly suitable for patients at risk due to prolonged anesthesia, peripheral vascular disease, or significant comorbidities.
